# Resting-State Connectivity of the Sustained Attention Network Correlates with Disease Duration in Idiopathic Generalized Epilepsy

**DOI:** 10.1371/journal.pone.0050359

**Published:** 2012-12-05

**Authors:** Mona Maneshi, Friederike Moeller, Firas Fahoum, Jean Gotman, Christophe Grova

**Affiliations:** 1 Montreal Neurological Institute and Hospital, McGill University, Montreal, QC, Canada; 2 Biomedical Engineering Department, McGill University, Montreal, QC, Canada; 3 Department of Neuropediatrics, Christian-Albrechts-University, Kiel, Germany; Charité University Medicine Berlin, Germany

## Abstract

**Introduction:**

In idiopathic generalized epilepsy (IGE), a normal electroencephalogram between generalized spike and wave (GSW) discharges is believed to reflect normal brain function. However, some studies indicate that even excluding GSW-related errors, IGE patients perform poorly on sustained attention task, the deficit being worse as a function of disease duration. We hypothesized that at least in a subset of structures which are normally involved in sustained attention, resting-state functional connectivity (FC) is different in IGE patients compared to controls and that some of the changes are related to disease duration.

**Method:**

Seeds were selected based on a sustained attention study in controls. Resting-state functional magnetic resonance imaging (fMRI) data was obtained from 14 IGE patients and 14 matched controls. After physiological noise removal, the mean time-series of each seed was used as a regressor in a general linear model to detect regions that showed correlation with the seed. In patients, duration factor was defined based on epilepsy duration. Between-group differences weighted by the duration factor were evaluated with mixed-effects model. Correlation was then evaluated in IGE patients between the FC, averaged over each significant cluster, and the duration factor.

**Results:**

Eight of 18 seeds showed significant difference in FC across groups. However, only for seeds in the medial superior frontal and precentral gyri and in the medial prefrontal area, average FC taken over significant clusters showed high correlation with the duration factor. These 3 seeds showed changes in FC respectively with the premotor and superior frontal gyrus, the dorsal premotor, and the supplementary motor area plus precentral gyrus.

**Conclusion:**

Alterations of FC in IGE patients are not limited to the frontal areas. However, as indicated by specificity analysis, patients with long history of disease show changes in FC mainly within the frontal areas.

## Introduction

Electroencephalogram (EEG) recording with generalized spike and wave (GSW) typically arising from normal background activity characterizes idiopathic generalized epilepsy (IGE). The main seizure types associated with IGE include absence, myoclonic, and tonic-clonic [1]. A large battery of psychological tests, including tests of personality, memory, problem solving and attention, have revealed that when matched for IQ, IGE patients performed poorly mainly on tests of visual sustained attention compared to healthy controls [2], [3], [4]. This reduced performance persisted even when errors associated with GSW bursts were excluded [5].

Sustained attention is defined as the ability to maintain a high vigilance level for a long time, allowing one to respond against presentation of infrequent and unpredicted events [6]. A widely used test of visual sustained attention is the Conners’ continuous performance test (CPT). In this test, also known as “not-X-CPT”, a list of letters is presented sequentially. While the target stimulus is the letter X, the task is to respond to non-target stimuli and to inhibit response to target stimuli [7]. Functional magnetic resonance imaging (fMRI) has been used in the past to identify those brain regions that are involved during visual CPT in healthy controls [8], [9], [10], [11]. Such studies provide a good opportunity to investigate possible alterations in the interaction of the corresponding brain regions in clinical population.

There are different approaches to investigate dysfunction of neural interactions. Among them, resting-state fMRI analysis is a suitable approach since it is not dependent on experimental design, subject compliance and training, making it of interest for studies of clinical populations. The way resting activity of the brain can be investigated using fMRI is functional connectivity analysis [12] and a hypothesis-driven approach to study functional connectivity is seed-based analysis [13]. This approach detects temporal correlations in spontaneous blood oxygen level dependent (BOLD) signal oscillations between a predefined seed and all the voxels in the brain. There are also exploratory approaches, such as independent component analysis (ICA) and clustering [14], [15], [16], [17], [18], which detect changes anywhere in the dataset without needing a prior definition of seeds. While exploratory approaches allow exploring networks in different types of diseases, their goal is not to investigate the changes in functional connectivity associated with specific regions involved in a particular function, as intended for the current study.

**Table 1:** Clinical Data.

Patient	Sex	Diagnosis	Age/onset	Seizuretypes	AED	DurationFactor
1	F	JAE	20/9	Abs, GTCS	LTG	0.5863
2	M	GTCS	38/19	Abs, GTCS	VPA, LTG	0.7706
3	M	JAE	41/10	Abs, GTCS	VPA, LTG	0.9843
4	M	JAE	36/15	Abs, GTCS	VPA	0.8101
5	F	JME	32/17	Abs, GTCS, jerks	VPA, CLB	0.6847
6	M	JME	22/16	GTCS, jerks	VPA	0.4330
7	M	JME	32/21	GTCS, jerks	LTG	0.5863
8	M	JME	47/15	GTCS, jerks	VPA, CLB, LEV	1.0000
9	F	EMA	22/4	EMA	LTG, CBZ	0.7500
10	F	EMA	27/4	EMA, GTCS	VPA	0.8478
11	F	JME	32/15	GTCS, jerks	VPA	0.7289
12	M	JME	20/19	GTCS	VPA	0.1768
13	F	JME	38/24	GTCS, jerks	CLB	0.6614
14	F	GTCS	43/12	GTCS	PHT	0.9843

IGE: idiopathic generalized epilepsy, not further defined; JAE: Juvenile absence epilepsy; EMA: eyelid myoclonia with absences; JME: Juvenile myoclonic epilepsy; Abs: absences; GTCS: generalized tonic clonic seizure; VPA: valproic acid; CLB: clobazam; LTG: lamotrigine; LEV: levetiracetam; PHT: phenytoin.

As the most explored difficulty of IGE patients is related to sustained attention [4], our assumption is to choose the seed locations in areas most involved during a sustained attention task in healthy controls. We used the results of the study published by Ogg et al. [19] to extract the seeds in our analysis. Their study included a large group of subjects and had a well-defined control condition. Most importantly, they found significant clusters that well covered all the areas reported by several other fMRI-CPT studies [8], [9], [10], [11]. Therefore, we ensured that our selection of seeds for functional connectivity analysis included all the brain areas that have been involved in the CPT task.

We performed a between-group functional connectivity analysis to specifically search for areas whose alterations in functional connectivity were correlated with clinical measures of IGE. We studied the differences between the average functional connectivity in controls and the duration-weighted average functional connectivity in patients. We chose the duration of epilepsy since it is an important factor in IGE, which correlates with the amount of cognitive decline [20], [21]. Then, we explored our results by evaluating the correlation between the amount of functional connectivity in each patient and the duration of epilepsy. There are other relevant clinical factors such as seizure frequency in a certain time period or the time interval from last ictal event. In this study we focus on duration of IGE, since we had this information for all our patients. We also tested for the effects of seizure frequency in a subset of our patients but we could not test for the effect of last ictal event. Our hypothesis is that at least in a subset of structures which are normally involved in sustained attention, the resting-state functional connectivity is different in IGE patients compared to healthy controls and that some of these changes are related to the disease duration.

**Table 2:** Seeds extracted based on the summary of regional activation during the CPT task relative to fixation.

Region	MNI X	MNI Y	MNI Z
**CPT>Fixation**			
R. Inf. frontal G.	52	20	−8
L. Inf. frontal G.	−58	10	30
R. Precentral G.	48	2	54
L. Precentral G.	−50	−6	54
R. Supramarginal G.	56	−38	24
R. Sup. temporal G.	50	8	−4
Med. Sup. frontal G.	0	12	46
L. Sup. cerebellum	−34	−66	−24
R. Sup. cerebellum	24	−60	−20
R. fusiform. G	40	−64	−16
R. Inferolateral occipital	46	−74	−12
**Fixation>CPT**			
R. Post. cingulate G.	2	−52	32
L. Inf. precuneus	−12	−58	20
L. Med. prefrontal	−6	48	−12
L. Inf. parietal lobule	−44	−70	46
R. Angular G.	48	−72	40
R. Postcentral	18	−34	64
R. Hippocampus	22	−12	−22

Inf.,Inferior; Med.,Medial; Sup.,Superior; Post.,Posterior; G.,Gyrus; R.,Right; L.,Left.

## Materials and Methods

### Ethics Statement

For this study, written informed consents as approved by the research ethics committee of the Montreal Neurological Institute and Hospital (MNI/H) were obtained from all the subjects. At its full board meeting of May 26, 2006, the Research Ethics Board (REB) of the MNI/H has endorsed the review of the project entitled: “Electrical, Metabolic and Structural Analysis of Human Epileptogenic Lesions” (Dr. J. Gotman being the principal investigator for this project). The REB of the MNI/H found this research to be acceptable for continuation at the McGill university healthcare centers (MUHC) and specifically approved this study. The REB of the MNI/H acts in conformity with standards set forth in the (US) Code of Federal Regulations governing human subjects research and functioning in a manner consistent with internationally accepted principles of good clinical practice.

**Table 3:** Summary of the duration-weighted group differences in resting-state FC.

**Seed Name**	**cluster** **Index**	**Number** **of voxels**	**Corrected** **p-value**	**Z-MAX**	**Z-MAX** **(X)**	**Z-MAX** **(Y)**	**Z-MAX** **(Z)**	**Brain Area corresponding to Z-max**
*R. Precentral G.	1	595	0.0015	3.93	−62	−26	−20	L. Inf. temporal. G
	2	1453	5.36E−07	3.93	−28	−12	56	L. premotor
**R. Sup. temporal G.**	1	852	1.97E−05	−3.67	28	−22	52	R. Precentral. G
	2	1912	6.64E−10	−4.07	−42	−20	54	L. Postcentral. G
*Med. Sup. frontal G.	1	825	9.05E−05	3.8	16	14	46	R. premotor
	2	854	6.73E−05	4.02	−14	10	46	L. premotor
	3	1043	1.05E−05	4.23	16	−34	56	R. Precentral. G
**R. fusiform. G**	1	1568	5.36E−07	4.25	22	−70	4	R. Med. occipital
**R. Inferolateral occipital**	1	1198	4.41E−06	4.33	4	−62	8	R. Med. occipital
*L. Med. prefrontal	1	625	0.0004	−3.74	36	−38	46	R. Postcentral. G
	2	2309	7.64E−11	−4.01	6	−10	62	L. Precentral. G & SMA
**L. Inf. parietal lobule**	1	1119	5.60E−06	−4.24	36	−88	−8	R. Inf. occipital. G
**R. Postcentral**	1	3775	1.54E−13	4.16	14	34	36	R. Med. frontal. G

Full results of seeds that showed significant functional connectivity change between the two groups (Z-threshold 2.7, p<0.05/18 corrected) for the contrast of patients minus controls. In cases where there were several peaks in each cluster, we only reported the highest one. Star sign indicates those seeds that not only their corresponding functional connectivity was significantly different between the two groups, but also the average functional connectivity, taken over significant clusters, was highly correlated with duration factor in patients (|*r*| >0.75, p<0.05/18). R: Right, L: Left, Med: Medial, Inf: Inferior, Sup: Superior, G: Gyrus.

**Figure 1:**
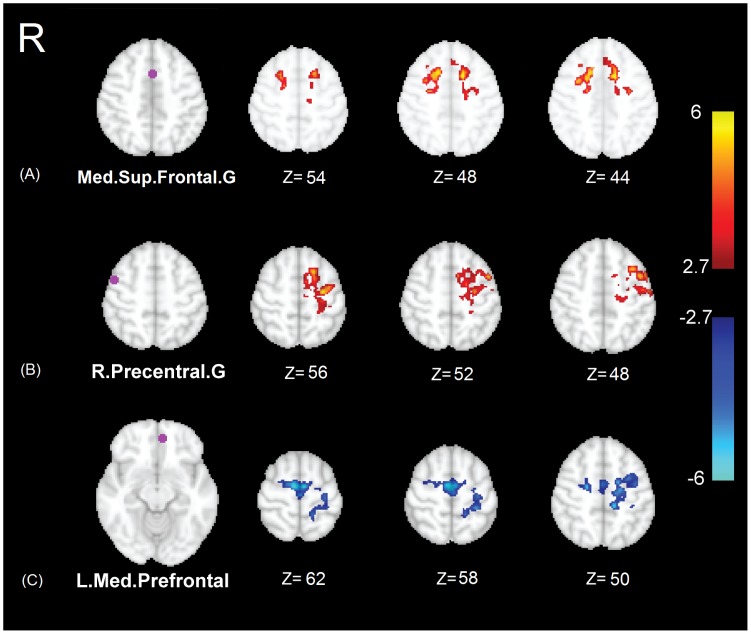
Results of significant duration-weighted group differences in functional connectivity (ΔFC). Left: seeds in purple and their names. Right: some selected slices illustrating group differences. The color-coded *Z*-score maps (p<0.05/18 corrected) show the results of alterations in functional connectivity in IGE patients compared to controls (for the contrast of patients minus controls). Positive functional connectivity is coded in red to yellow, while negative in blue to white. Note that Figure 1 only shows those clusters whose functional connectivity in IGE patients significantly correlates with the duration factor (*|r|* >0.75, *p*<0.05/18).

**Figure 2:**
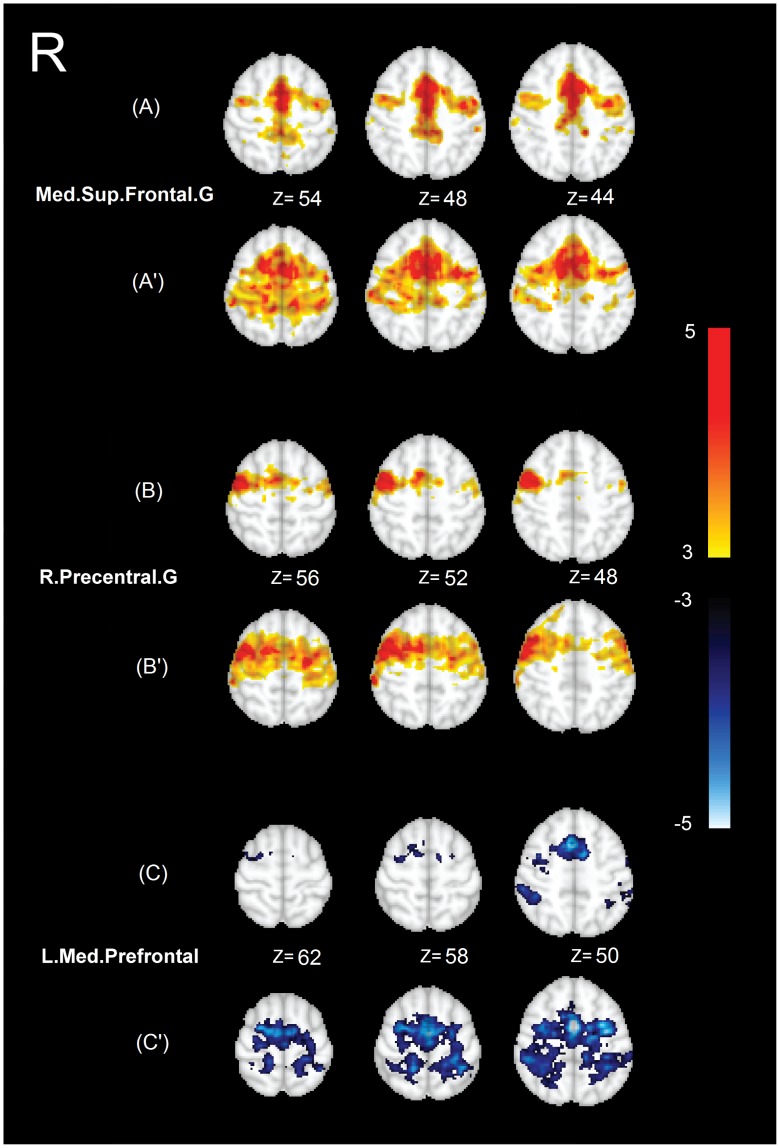
Results of average functional connectivity within each group of subjects for the seeds reported in Figure 1. Left: seeds names. Right: The same selected slices illustrating average functional connectivity within controls (A–C), and average functional connectivity within patients (A′–C′), Color-coded statistical Z-score maps (p<0.05/18 corrected) showing a positive (coded in yellow to red) and a negative (coded in blue to white) functional connectivity.

**Figure 3:**
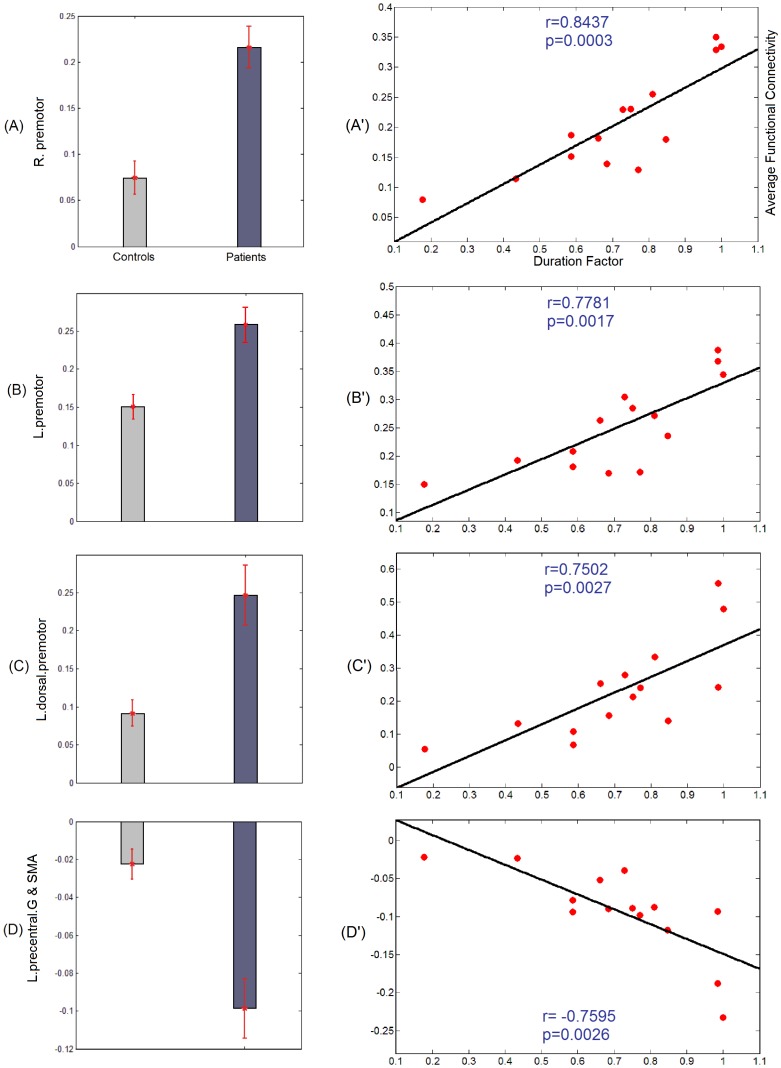
Results of correlation analysis for the seeds reported in Figure 1. (A–D): Bar diagrams illustrating average functional connectivity within each group over the clusters of significant differences reported in Figure 1. Figure 3 (A, B) corresponding to the seed in medial superior frontal gyri, respectively illustrate the average functional connectivity within each group over clusters in the right and left premotor area. Figure 3 C corresponding to the seed in right precentral gyrus, illustrates the average functional connectivity within each group over a cluster in the left dorsal premotor area. Figure 3 D corresponding to the seed in the left medial prefrontal area, illustrates the average functional connectivity within each group over a cluster in the left precentral gyrus and the supplementary motor area. (A′–D′): average functional connectivity within patients for the clusters discussed in (A–D) as a function of the duration factor. The black line shows the fitted line for correlation analysis. Correlation coefficient *r* and *p*-value are shown for each correlation analysis.

### Subjects

Our EEG-fMRI dataset of patients scanned at 3 Tesla contained 14 patients with IGE (aged 33+/−9 years, 7 males, and 7 females). All patients were taking medication at the time of study and they did not stop it for the purpose of scanning. The study was approved by the research ethics board of the Montreal Neurological Institute & Hospital (“MNI/H REB”) and subjects participated in the research after giving written informed consent. Patients inclusion criteria were: a) having at least two runs without GSW during the scan (fMRI recording included 6–14 six-minute runs), b) wakefulness proven by EEG recording during these runs, c) motion of less than 1 mm as determined by the realignment of the preprocessing (see section 2.5, preprocessing step 4). Table 1 gives the demographic and clinical characteristics of all patients. Fourteen age and sex-matched healthy controls (aged 33+/−8 years) were scanned using the same EEG-fMRI protocol, fulfilling inclusion criteria b) and c). There was no significant difference between the age distributions of the two groups (sign test, p>0.05).

### EEG Acquisition

The EEG acquisition was performed using 25 MR compatible electrodes (Ag/AgCl) placed on the scalp using the 10–20 (21 usual electrodes without Fpz and Oz, reference at FCz) and 10–10 (F9, T9, P9, F10, T10 and P10) electrode placement systems. Two electrodes located on the back recorded the electrocardiogram (ECG). To minimize movement artifacts and for the patient’s comfort, the head was immobilized using a pillow filled with foam microspheres (Siemens, Germany). Data were transmitted from a BrainAmp amplifier (Brain Products, Munich, Germany, 5 kHz sampling rate) via an optic fiber cable to the EEG monitoring computer located outside the scanner room.

### fMRI Acquisition

Functional images were continuously acquired using a 3T MR scanner (Siemens Trio, Germany). A T1-weighted anatomical acquisition was first done (1 mm slice thickness, 256×256 matrix; TE = 7.4 ms and TR = 23 ms; flip angle 30°) and used for superposition with the functional images and inter-subject group co-registration. IGE patients had 7–14 and the healthy controls had 4 runs of 6 min each. However, only a subset of those runs was selected based on the mentioned inclusion criteria. In addition, to have the same number of runs for all subjects, only 2 runs were included at the end for each subject. The functional data were acquired using a T2^*^-weighted EPI sequence (5×5×5 mm voxels, 25 slices, 64×64 matrix; TE = 30 ms and TR = 1750 ms; flip angle 90°). No sedation was given and resting-state was defined as a state of relaxed wakefulness when subjects have their eyes closed and are instructed to refrain from any structured thoughts.

### EEG Processing

The Brain Vision Analyzer software (Brain Products, Munich, Germany) was used for off-line correction of the gradient artifact and filtering of the EEG signal. This software uses the method described by Allen and colleagues [22]. A 50 Hz low-pass filter was also applied to remove remaining high-frequency artifact. The ballistocardiogram (BCG) artifact was removed by independent component analysis [23], [24]. A neurologist reviewed the EEG recordings and made sure that the selected runs in IGE patients were GSW free and that the patients and controls were awake during these runs. Figure S1 provides a sample of such data in selected patients.

### Seed-based Functional Connectivity Group Analysis

Data processing was carried out using FMRIB Software Library (FSL), www.fmrib.ox.ac.uk, Oxford U.K., FSL version 4.1 [25], [26]. The following preprocessing steps were applied: (1) removal of the first two volumes of each scan to allow for equilibrium magnetization, (2) slice timing correction using Fourier-space time-series phase-shifting, (3) non-brain tissue removal [27], (4) motion correction using a 6-parameter linear transformation using a maximization of the correlation ratio (default settings of FSL) [28], (5) intensity normalization of all volumes of each run as implemented in FSL (6) spatial smoothing using a Gaussian kernel with 6 mm full width at half maximum (FWHM), (7) high-pass temporal filtering with cut off frequency of 0.01 Hz. To achieve the transformation between the low-resolution functional data and the average standard space (MNI152: average T1 brain image constructed from 152 normal subjects [29]), we performed two transformations. The first was from the low resolution EPI image to the T1-weighted structural image (using a 7 degrees of freedom affine transformation), and the second was from T1-weighted structural image to the average standard space (using a 12 degrees of freedom linear affine transformation, voxel size = 2×2×2 mm).

Removing the physiological noise (cardiac and respiratory related signals) has a great impact on the results of functional connectivity analysis at rest [30], [31], [32]. We used a method similar to that of Shehzad et al. [33], [34], by employing the average signals taken over sections of white matter (WM), cerebro-spinal fluid (CSF), and the whole brain (the so-called global signal) as nuisance regressors. In order to do this, we segmented each individual’s high-resolution T1-weighted image, using an automatic segmentation software [35] providing tissue probabilistic maps for Grey Matter, White Matter and CSF. The resulting probabilistic tissue maps of WM and CSF images were then thresholded to identify masks of voxels showing more than 80% probability of belonging to the corresponding tissue class. Each thresholded mask was then applied to that individual’s time series and the mean time series was calculated by averaging the time series from all the voxels within the mask. The global signal accounts for several potential sources of physiological noise assuming that fMRI experiments are concerned with local changes in neuronal activity and that global signals represent uninteresting sources of noise [36], [37]. Nine nuisance regressors were thus considered for the first general linear model (GLM) (WM, CSF, global signal and 6 motion parameters: x, y, and z translations and rotations obtained from motion correction step in preprocessing).

Thus, for each run of each individual, a GLM analysis was carried out using the time series of nuisance signals [38]. The purpose of this first regression analysis was to model the nuisance signals as much as possible so that these sources of artifact can be removed from the data. To extract the seeds’ time series, the MNI coordinates of both activation and deactivation peaks reported in the study of Ogg et al. [19] were considered. The activation peaks correspond to regions activated more during CPT than fixation (CPT>Fixation), and the deactivation peaks correspond to regions with a higher activity in fixation compared to CPT (Fixation>CPT). In cases where they had reported several clusters in one region, we considered the peak corresponding to the largest cluster. Overall, 11 activation-related and 7 deactivation-related seeds were selected (Table 2).

In order to obtain BOLD time-series for each seed in every run, first a spherical mask (radius = 6 mm) around the seed in the standard space was defined. Then this mask was resampled into the low-resolution functional space and finally, the mean time-series over the mask was calculated. For each run of each individual, the mean time-series of the seed was then used as a regressor in a GLM model to find voxels that show correlation with that seed. For each seed, the two runs of each individual were combined using fixed effects model. The resulting functional connectivity map for each individual was then transformed to the MNI space. This analysis was followed by between-group analysis performed using a mixed-effects model [39]. This GLM analysis included the duration factor, explained below, as a regressor.

The duration of epilepsy was obtained for each patient as a clinical measure [20]. Since the original duration values (Table 1) are count data in terms of number of years of having IGE, the square root transformation was applied to make their distribution closer to a Gaussian distribution [40]. We then scaled these values to range between 0 and 1 (note that scaling by a constant value does not change the GLM results). We named these normalized values *duration factors*. We performed between-groups analyses using the mixed-effects model implemented in FSL [39]. To incorporate the clinical measures in the group-level analysis we used a similar approach as explained in Vahdat et al. [41]. We considered two regressors in group-level GLM: one modeling the average of healthy control subjects, and the other one modeling a weighted average of epileptic patients based on duration factor. Then, the contrast of interest was defined as the difference between these two regressors. This analysis increases the sensitivity for detecting changes between the two groups, which are related to the clinical measures of epilepsy duration.

Corrections for multiple comparisons at the cluster level was carried out based on random field theory as implemented in FSL [39] (threshold |*Z*| >2.7; cluster significance: *p*<0.05, corrected). To correct for multiple seeds, we identified as statistically significant the clusters with a probability level lower than *p* = 0.05/18 = 0.0028 (Bonferroni correction, 18 being the number of seeds). This between-subjects analysis produced thresholded *Z* score maps of activity associated with each seed. The positive values in the *Z*-map corresponded to areas where functional connectivity was elevated in patients compared to controls. The negative values corresponded to areas where functional connectivity was reduced in patients compared to controls. The anatomical regions were labeled according to the Harvard-Oxford cortical and subcortical [42], and Juelich histological atlases [43]. In the patient group, we calculated the Pearson correlation between the average functional connectivity taken over each significant cluster and the duration factor. Correlation coefficients larger than 0.75 (|*r*| >0.75, corresponding to *p*<0.05/18, corrected for multiple comparisons) were considered as statistically significant in our analysis.

In addition, we extracted seizure frequency (number of seizures in the last 2 years before the scan date), and time interval from the last ictal event as two extra clinical measures. Similar to the approach used to calculate the duration factor from duration of epilepsy, seizure frequency values were converted into a *frequency factor* calculated for each patient. However, only 8 patients had non-zero values for this measure. Consequently, they were compared with 8 age- and gender-matched healthy controls in the subsequent GLM analysis. This regression analysis was similar to the one previously proposed, replacing duration factor by frequency factor. As seizures were at least 1 month away for all our patients that we had information about, we did not include time from last ictal event as a factor in our analysis (see File S1 and Table S1).

## Results

Using the seed coordinates reported in Table 2, we tested the difference in functional connectivity between IGE patients and healthy controls with respect to the duration factor. Eight out of 18 seeds showed a significant difference weighted by duration factor, in functional connectivity across groups. Among them, seeds in right precentral, superior temporal, fusiform, inferolateral occipital, and in medial superior frontal gyri correspond to the activated regions during CPT reported by Ogg et al., [19]. In contrast, seeds in left medial prefrontal, inferior parietal lobule, and in right postcentral gyrus correspond to the deactivated regions during CPT. All significant clusters (*p*<0.05/18, corrected for multiple comparisons) are summarized in Table 3. This table reports the number of voxels in each cluster, cluster’s *p*-values, peaks’ coordinates and *Z*-values, and the related anatomical labels.

Among the 13 clusters showing significant changes of functional connectivity in IGE patients compared to controls as reported in Table 3, only 4 showed a significant correlation with the duration factor (correlation coefficient |*r*| >0.75, p<0.05/18 corrected for multiple comparisons). Among them, two clusters corresponded to seed in the medial superior frontal gyri, and two corresponded to seeds in the right precentral gyrus and left medial prefrontal area. Figures 1, 2, 3 illustrate the results of analysis related to each of these clusters. Note that Figure 1 only shows those clusters whose functional connectivity in IGE patients significantly correlates with the duration factor. The seed in the medial superior frontal gyri showed an elevation of functional connectivity in patients with bilateral premotor and superior frontal gyrus, as shown in Figure 1 A. The seed in right precentral gyrus showed an elevation of functional connectivity in patients with the left dorsal premotor area, as illustrated in Figure 1 B. Also the seed in left medial prefrontal area showed an elevation in the absolute value of functional connectivity in patients with the left precentral gyrus and supplementary motor area (SMA), shown in Figure 1 C.

In Figure 2, we illustrated the maps of average functional connectivity for each group separately. The functional connectivity maps are shown for the same seeds as in Figure 1. Figures 2 A–C show the average functional connectivity maps in healthy controls, and A′–C′ demonstrate similar maps in IGE patients. As shown in this figure, in all cases the functional connectivity maps are spatially more spread in IGE patients compared to healthy controls. Specifically, the pattern of connectivity for the seed in right precentral gyrus is bilateral, as opposed to healthy controls. Also, the seed in the medial prefrontal area shows a broad pattern of negative functional connectivity with the sensorimotor cortex in IGE patients. Figure 2 A illustrates the functional connectivity map within the working memory and attention-related areas [44], Figure 2 B the inter-hemispheric functional connectivity map of the motor network [13], and Figure 2 C the negative functional connectivity map between a major node of the default mode network (DMN) and sensorimotor areas [45].

We tested for the correlation between the functional connectivity, averaged over each cluster with significant group differences, and the duration factor. This analysis resulted in a pattern of correlations, which is included in Figure 3. The mean and standard errors of functional connectivity within IGE and healthy groups in the 4 clusters (2 clusters corresponding to the seed in the medial superior frontal gyri, one to the seed in the right precentral gyrus, and one to the seed in the left medial prefrontal area) are illustrated in Figure 3 A–D. Also, results of IGE patients showing significant correlations (*|r|* >0.75, *p*<0.05/18) between the average functional connectivity, taken over significant clusters, and the duration factor are illustrated in Figure 3 A′–D′. For instance, Figure 3 A′ shows that in IGE patients, the elevated functional connectivity between the seed in the medial superior frontal gyri and the cluster in the left dorsal premotor area is significantly correlated (*r* = 0.84, *p* = 0.0003) with their duration factor. The strong correlation emphasizes the strong association between the functional connectivity values and duration factor on a per subject basis.

For all the seeds reported in Table 2, we also tested the changes of functional connectivity with respect to the frequency factor from a group subset composed of 8 patients and 8 corresponding healthy controls. Out of 18 tested seeds, only one in the right superior temporal gyrus showed reduction of functional connectivity in patients, with two clusters in the precentral and postcentral gyri (Figure S2). However, for these two clusters the average functional connectivity in IGE patients was not significantly correlated with the frequency factor.

In order to test for the specificity of our analysis in finding alterations in functional connectivity across groups with respect to the duration factor, we analyzed additional seeds extracted from consistently reported resting-state networks. We assessed functional connectivity associated with three seeds in the task positive network including intraparietal sulcus [(−25, −61 47)], the frontal eye field region [(25, −16, 54)], and the middle temporal region [(45, 69, 2)] [46]. Furthermore, we investigated seeds in the executive network [(−42, 34, 20), (44, 36, 20); left and right dorsolateral prefrontal cortices], the salience network [(38, 22, −10); insular cortex], the motor network [(25, −16, 54); precentral gyrus], the auditory network [(−48, −24, 9); Heschl’s gyrus], and the visual network [(−2, −82, 4); primary visual cortex] [47]. In summary, none of these seeds showed any significant duration-weighted difference (threshold |*Z*| >2.7, *p*<0.002 corrected for multiple comparisons) in functional connectivity across groups. The only differences were observed for seeds located in visual and motor networks (consistent with the results listed in Table 3). However, in the patient group, the average functional connectivity taken over each significant cluster did not show any significant correlation (threshold |*r*| >0.75, *p*<0.002) with the duration factor.

## Discussion

We used seed-based functional connectivity analysis to study resting-state brain activity in patients with IGE. The seeds were selected in regions normally involved in sustained attention and we demonstrated that the resting-state functional connectivity of a subset of these structures is different in IGE patients compared to healthy controls. Eight of the 18 selected seeds showed differences in functional connectivity across groups. Among these 8 seeds, 3 showed a strong correlation between the disrupted amount of functional connectivity and IGE duration factor. These seeds were in the medial superior frontal gyri, the right precentral gyrus, and the left medial prefrontal area. The clusters showing altered functional connectivity with these 3 seeds were respectively located in bilateral premotor and superior frontal gyrus, in left dorsal premotor, and in SMA and left precentral gyrus. Our findings were specific to these regions as none of the 9 additionally tested seeds, extracted from consistently reported resting-state networks, resulted in any significant correlation between the duration factor and the functional connectivity in patients.

In summary, our results indicate that alterations of functional connectivity in IGE patients are not limited to the frontal areas, but also include other areas in the parietal and occipital lobes (see Table 3). However, only the frontal areas show functional connectivity alterations that are highly correlated with the duration factor. In other words, in several frontal lobe areas, patients with a longer history of disease showed greater alterations in functional connectivity compared to those with a shorter history.

We found that in IGE patients, the seeds in different frontal areas show elevated functional connectivity with extended brain areas; if the functional connectivity value was positive in controls, it became more positive in patients (seeds in medial superior frontal and right precentral gyri), and if it was negative, it became more negative (seed in the left medial prefrontal area). The former pattern suggests that the networks involved in working memory and motor-planning are hyperactive in patients, while the latter suggests that a major node of the DMN shows an abnormally elevated anti-correlation with the frontal motor areas in patients. It might be that in IGE patients as a result of GSW recurrence some neural connections have been facilitated [48], [49], which in turn led to elevation in magnitude and extension in functional connectivity. Specifically, in the frontal areas the degree of elevation is correlated with epilepsy duration.

In IGE patients, subtle changes in gray matter thickness and white matter integrity in areas including medial superior frontal and precentral gyri, and prefrontal cortex have been reported when compared to healthy subjects [50], [51], [52], [53]. In some cases, the amount of structural changes was correlated with disease duration [52]. These regions are also highly involved during GSWs [54], [55], [56], [57], [58]. We also found alterations in resting-state functional connectivity in the same regions. The observed pattern of elevation and extension in functional connectivity might be caused by the aforementioned structural changes, or the long term GSWs, or both.

Several neuropsychological studies in IGE patients have reported deficits in frontal lobe executive and cognitive functions [20], [59], [60]. In a CPT study in absence epilepsy, all behavioral impairment measures were associated with reduced medial superior frontal cortex activation [61]. Then by extracting seed locations from this region, resting-state functional connectivity analysis revealed impaired connectivity with the right anterior insula/frontal operculum. In another cognitive fMRI study, the motor system showed hyper-connectivity in patients with juvenile myoclonic epilepsy (JME) [62]. They reported that with increasing cognitive demand, patients showed increasing co-activation of the primary motor cortex and supplementary motor area. This is in agreement with our results showing elevated pattern of functional connectivity between the motor and premotor areas. Also, changes related to the DMN have been reported in resting-state studies of IGE patients [63], [64], [65]. In a study by Wang et al. [63], changes in DMN functional connectivity were correlated with seizure duration. In other studies [64], [65], decreased integration within the DMN was found in the IGE group. These studies generally reported changes in correlations within the DMN, while we found changes in anti-correlation between a major node of the DMN and the sensorimotor network.

Although we are aware that artificial anti-correlation may be introduced by regressing out the global signal, this is still an open issue. As an example, Murphy et al. reported that global signal removal almost doubled connection specificity [66]. Similarly, other studies demonstrated that there is an enhancement in the detection of system-specific correlations and an improvement in the correspondence between resting-state correlations and anatomy when the global signal is regressed out [67], [68]. In any case, this issue may not concern our study, since we were doing comparison between patients and controls.

The choice of resting-state fMRI helped us to study multiple cortical systems at the same time, in contrast to task-activation analyses which require dedicated data acquisitions for each function that one is attempting to localize [69]. Patients were on different medications and this could be a confounding factor between patients and controls. Unfortunately, it is almost impossible to dissociate the long term effect of medication from the effect of disease when studying patients with a long duration of epilepsy. The reason is that the vast majority of epilepsy patients take a combination of different medications since the onset of their disease. Our results reflect the state of the brain of epileptic patients, which may relate to long- standing epilepsy, long-standing medication, both in combination, or a common cause for epilepsy and attention problems. However, a causal relationship cannot be inferred from this analysis. Given the number of patients, this study does not allow us to separate the effect of different medications. Our findings provide a functional basis for impaired interictal functional connectivity in IGE patients in relation to the clinical measure of epilepsy duration, which may allow the development of improved treatments targeted at these areas.

The conclusion that the facilitated neural connections within frontal regions detected by fMRI can be a biomarker of IGE requires more investigations from different techniques to be done in future.

## Supporting Information

Figure S1 **A sample of awake baseline EEG data.** Sample of EEG data in the awake state illustrated in bipolar (A) and in referential montage (B).
(TIF)

Figure S2 **Results of significant frequency-weighted group differences in functional connectivity in a group subset composed of 8 patients and 8 corresponding healthy controls.** Left: seed in the right superior temporal gyrus shown in purple. Right: some selected slices illustrating group differences. The color-coded *Z*-score maps (p<0.05/18 corrected) show the results of alterations in functional connectivity in IGE patients compared to controls (for the contrast of patients minus controls). Negative functional connectivity is coded in blue to white.
(TIF)

Table S1 Extra clinical measures. Values of frequency factor and time interval from last ictal event for the patients reported in Table 1.
(DOC)

File S1 Testing the effect of seizure frequency and time interval from the last ictal event on functional connectivity in IGE patients.
(DOC)
