# Supplementary figures and images for: Resting-State Connectivity of the Sustained Attention Network Correlates with Disease Duration in Idiopathic Generalized Epilepsy
Source: PLoS One. 2012 Dec 5;7(12):e50359. doi: 10.1371/journal.pone.0050359 (PMC3515589; doi:10.1371/journal.pone.0050359)

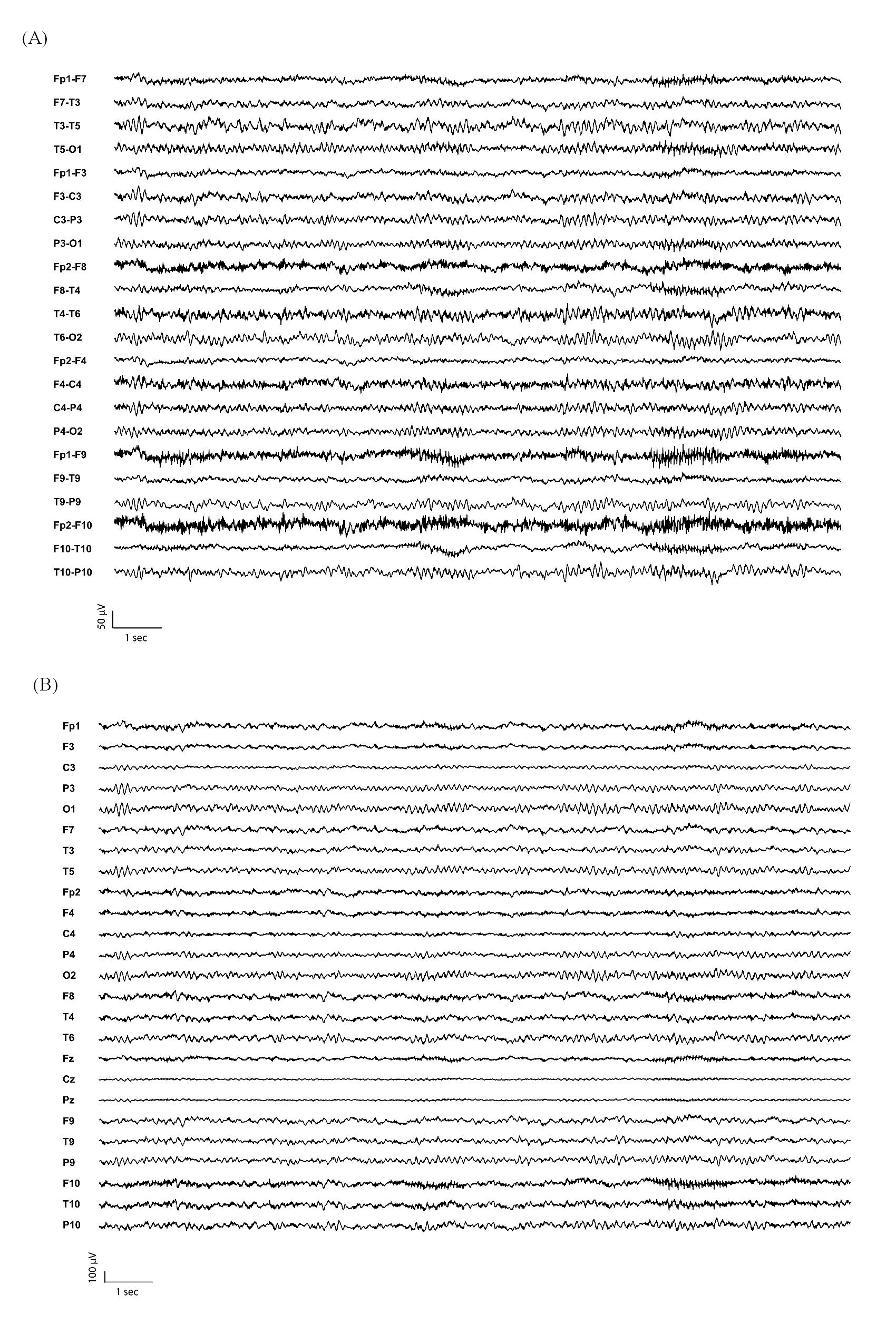

Supplement: Figure S1 — A sample of awake baseline EEG data. Sample of EEG data in the awake state illustrated in bipolar (A) and in referential montage (B). (TIF) [file pone.0050359.s001.tif]

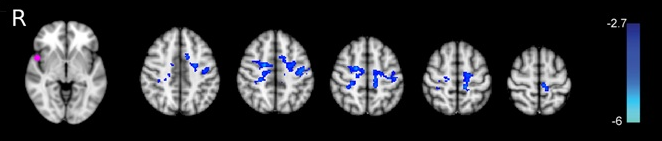

Supplement: Figure S2 — Results of significant frequency-weighted group differences in functional connectivity in a group subset composed of 8 patients and 8 corresponding healthy controls. Left: seed in the right superior temporal gyrus shown in purple. Right: some selected slices illustrating group differences. The color-coded Z-score maps (p<0.05/18 corrected) show the results of alterations in functional connectivity in IGE patients compared to controls (for the contrast of patients minus controls). Negative functional connectivity is coded in blue to white. (TIF) [file pone.0050359.s002.tif]
