# Supplementary material for: Resting-State Connectivity of the Sustained Attention Network Correlates with Disease Duration in Idiopathic Generalized Epilepsy
Source: PLoS One. 2012 Dec 5;7(12):e50359. doi: 10.1371/journal.pone.0050359 (PMC3515589; doi:10.1371/journal.pone.0050359)
Supplement: Table S1 — Extra clinical measures. Values of frequency factor and time interval from last ictal event for the patients reported in Table 1. (DOC) [file pone.0050359.s003.doc]

| **Patient** | **Frequency**  **factor** | **Last**  **seizure** |
| --- | --- | --- |
| 1 | 0.3536 | Unknown |
| 2 | 1.0000 | Unknown |
| 3 | 1.0000 | 1 month |
| 4 | 0.0000 | Unknown |
| 5 | 0.0000 | 2 years |
| 6 | 0.0000 | 5 years |
| 7 | 0.2500 | 11 months |
| 8 | 0.5590 | 5 months |
| 9 | 0.0000 | 3 years |
| 10 | 0.0000 | 3 years |
| 11 | 0.2500 | Unknown |
| 12 | 0.4330 | 3 months |
| 13 | 0.0000 | 10 years |
| 14 | 0.2500 | 4 months |
