# Supplementary material for: Resting-State Connectivity of the Sustained Attention Network Correlates with Disease Duration in Idiopathic Generalized Epilepsy
Source: PLoS One. 2012 Dec 5;7(12):e50359. doi: 10.1371/journal.pone.0050359 (PMC3515589; doi:10.1371/journal.pone.0050359)
Supplement: File S1 — Testing the effect of seizure frequency and time interval from the last ictal event on functional connectivity in IGE patients. (DOC) [file pone.0050359.s004.doc]

**Supporting Information**

In addition to epilepsy duration, we extracted seizure frequency (number of seizures in the last 2 years before the scan date), and time interval from the last ictal event as two extra clinical measures. Regarding the seizure frequency, patients with no seizures in the past 2 years (6 patients) were excluded from the subsequent analysis, as this measure was not able to differentiate them from healthy controls. Since the original seizure frequency values (Table S1) are count data, the square root transformation was applied to make their distribution closer to a Gaussian distribution. We then scaled these values to range between 0 and 1. We named these normalized values the frequency factor. The subsequent analysis (on the 8 remaining patients and the corresponding age- and gender-matched controls) was similar to that explained in the method section for the duration factor. In the patient group, we also evaluated the correlation between the average functional connectivity taken over each significant cluster and the frequency factor. Correlation coefficients larger than 0.75 (|*r*| > 0.75, corresponding to p < 0.05/18, corrected for multiple comparisons) were considered as statistically significant in this analysis. Testing all the seeds reported in Table 2, only one seed in the right superior temporal gyrus showed reduction of functional connectivity in patients, with two clusters in the precentral and postcentral gyri (Figure S2). However, for these two clusters the average functional connectivity in IGE patients was not significantly correlated with the frequency factor (r = -0.6248 for the left cluster and -0.6828 for the right one).

Regarding the time from last ictal event, 5 out of 14 patients had an interval longer than 2 years, 5 had an interval between 1 month and 2 years, and 4 had unknown last ictal event. As seizures were at least 1 month away for all our patients that we had information about, we did not include time from last ictal event as a factor in our analysis.
